# 

**Published:** 1891-11-21

**Authors:** 


PRICE ONE PENNY.

No. 269. Vol. XI.—November 21st, 1891.

Registered at the General Post Office as a Newspaper for Transmission in the United Kingdom and Abroad.]

WITH EXTRA NURSING SUPPLEMENT.
Per Annum, 6s. 6d., post free.
Monthly Parts, 6d.; post free, 8d.
Ditto per Annum, 8s., post free.

### THE HOSPITAL

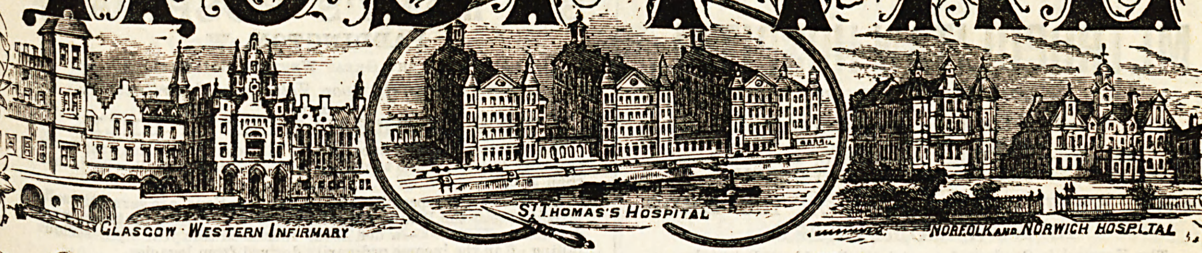

A WEEKLY JOURNAL OF

### Science, Medicine, Nursing, and Philanthropy.

SATURDAY, NOVEMBER 21st, 1891.

**America and Her Hospital Workers** . . . . . 85

**Passing Topics.**—Survivors . . . . . . . . . 86

**The Doctor's Armchair.**—The Sick in Uncivilised Lands . 87

**Some Scientific Aspects of Insanity. III.**—Nerve Tissue 88

**Hospitals of the North-West. II.**—Vancouver . . . 89

**Notes and News** . . . . . . . . . 90

**General Charities** . . . . . . . . . 91

**Scraps and Gleanings** . . . . . . . . 91

**Annotations.**—Think of It—Genius and Insanity—A Venerable Volume . . . . . . . . . 92

**The Practitioner's Mirror and Retrospect—**

Medicine.—Some Points in the Treatment of Dyspepsia . 93

Surgery.—On the Question of Excision in Tubercular Hip Disease . . . . . . . . . . . 94

Practitioner's Bookshelf.—Arcana Fairfaxiana . . . 95

**Editor's Letter Box.**—Professor Germane and the Medical Experts . . . . . . . . . . . 93

**The Story oi the Insane from Year to Year** . . 96

**The Student of Medicine.**—Appointments—Vacancies—Notes from the Schools—Pass Lists—Athletics

EXTRA SUPPLEMENT.—THE NURSING MIRROR.

**En Passant.**—Northamptonshire Institution—A Nurses' Home for Aberdeen—Belfast News—Music as a Soporific—Thermometers—Short Items—Daily Nurses . . . . xliii

**On Nurses and their Dress** . . . . . . . xliv

**A Nurse in Natal** . . . . . . . . . xliv

**A Bed for a ick Nurse** . . . . . . . . xlv

**For Reading to the Sick**—The Rainbow . . . . xlv

**Our Indian Letter** . . . . . . . . . xlvi

**The Nurses' Bookshelf.**—A Manual for Nurses—Two Old Friends . . . . . . . . . . . xlvii

**Appointments—Presentation** . . . . . . xlvii

**Notes and Queries** . . . . . . . . . xlvii

**"Dr. Sutters"** . . . . . . . . . . xlviii

**Amusements and Relaxation** . . . . . . xlviii

